# Behavioural specialization and learning in social networks

**DOI:** 10.1098/rspb.2022.0954

**Published:** 2022-08-10

**Authors:** Olof Leimar, Sasha R. X. Dall, Alasdair I. Houston, John M. McNamara

**Affiliations:** ^1^ Department of Zoology, Stockholm University, 106 91 Stockholm, Sweden; ^2^ Centre for Ecology and Conservation, University of Exeter, Penryn TR10 9FE, UK; ^3^ School of Biological Sciences, University of Bristol, Bristol BS8 1TQ, UK; ^4^ School of Mathematics, University of Bristol, Bristol BS8 1UG, UK

**Keywords:** behavioural consistency, animal personality, reinforcement learning, game theory

## Abstract

Interactions in social groups can promote behavioural specialization. One way this can happen is when individuals engage in activities with two behavioural options and learn which option to choose. We analyse interactions in groups where individuals learn from playing games with two actions and negatively frequency-dependent payoffs, such as producer–scrounger, caller–satellite, or hawk–dove games. Group members are placed in social networks, characterized by the group size and the number of neighbours to interact with, ranging from just a few neighbours to interactions between all group members. The networks we analyse include ring lattices and the much-studied small-world networks. By implementing two basic reinforcement-learning approaches, action–value learning and actor–critic learning, in different games, we find that individuals often show behavioural specialization. Specialization develops more rapidly when there are few neighbours in a network and when learning rates are high. There can be learned specialization also with many neighbours, but we show that, for action–value learning, behavioural consistency over time is higher with a smaller number of neighbours. We conclude that frequency-dependent competition for resources is a main driver of specialization. We discuss our theoretical results in relation to experimental and field observations of behavioural specialization in social situations.

## 1. Introduction

The issue of why individuals differ in behavioural tendencies has received much attention in recent years [1–3], with a focus on genetic or other differences emerging early in development. One influential idea is that frequency dependence can promote specialization [4]. Here, we explore the possibility that learning with frequency-dependent rewards, such as rewards from playing games, can give rise to specialization.

Early in the development of game theory in biology it was found that there can be asymmetric evolutionarily stable strategies (ESSs), with the ‘bourgeois’ ESS for the hawk–dove game as a well-known example [5,6]. In this game two individuals interact and the ESS is polarized, in the sense that one player uses hawk and the other dove. It turns out that there are similar ESSs for group sizes larger than two, such that players polarize into using different behavioural options [7]. The selection favouring polarization is stronger in smaller groups. Here, we extend the idea of behavioural specialization to groups interacting in a social network, where the group size might be large but the number of network neighbours of an individual could be small. We focus on learning leading to specialization, because social interactions are often repeated in a group and persist over times long enough for learning to be important.

The idea that frequency-dependent learning leads to specialization was introduced some time ago [8], with producer–scrounger relations [9] as a possible example. Recent foraging experiments have demonstrated that negatively frequency-dependent learning can result in behavioural diversity, with preferences becoming established after 25–50 foraging experiences per individual [10], which corresponds to rather fast learning. Producer–scrounger experiments with birds also indicate that behavioural specialization involves learning [11] and that behaviour is consistent over time if the social environment (the flock mates) is constant, but tends to change in new social environments [12]. Stable producer–scrounger relations are also found in bats that live in large groups, but interact when foraging with a small number of other individuals, thus forming a social network [13].

The general idea of frequency-dependent learning in social groups is thus well established and has experimental support, but up to now it is not known how the social environment, in particular, the number of network neighbours, influences the rate of establishment and the temporal stability of behavioural specialization. Our aim here is to examine these questions, using game-theory models of groups of individuals that learn, based on rewards (i.e. payoffs), which actions to prefer when interacting with neighbours in a social network. In addition to the producer–scrounger game [9,14,15], where individuals have the options to produce (i.e. search for a food source) or to scrounge (i.e. attempt to exploit food sources found by producers), we also study a caller–satellite game [16–18] and the hawk–dove game [6,19].

Calling and acting as satellite are male behavioural options in species in which males call to attract females or, alternatively, act as satellites to nearby callers, attempting to intercept approaching females. In anurans, calling involves a form of male–male competition [20], so that males can be seen as interacting with neighbours in a social network [21], and the situation could be similar in other species with calling males.

The hawk–dove game is frequently used to examine contests between individuals, but it gives a highly schematic of such behaviour. Contests in social groups often produce dominance hierarchies with individual recognition, but there may be examples of fights in social groups with limited or no individual recognition, such as in some species of crickets [22,23], where learning to prefer hawk versus dove in aggressive interactions, which corresponds to dominant versus subordinate behaviour, can provide a modelling starting point. Also, for repeated hawk–dove interactions between two individuals, a reinforcement-learning model showed polarization, one individual using hawk and the other dove [24].

In each of the games we study, we idealize the situation by assuming that group members do not differ in traits like learning, foraging or fighting abilities, in order to focus on the particular effects of frequency-dependent learning. For learning, we use reinforcement-learning approaches (action–value learning and actor–critic learning) that encapsulate basic learning concepts from animal psychology [25]. Action–value learning is the simplest of these and is an implementation of the Rescorla–Wagner model for operant conditioning. The learned probabilities of choosing actions are based on differences in estimates of the value (expected reward) from using an action.

## 2. Methods

**Figure 1.**
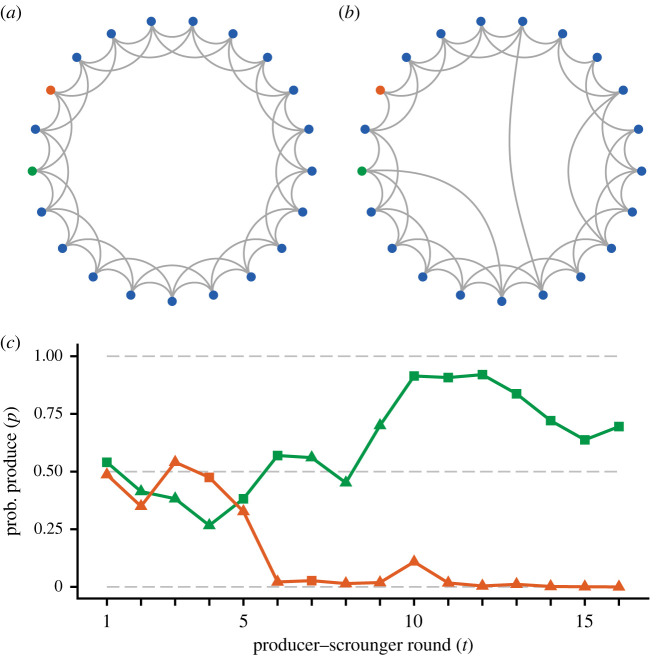
Illustration of networks with social interactions. (*a*,*b*) Coloured points represent individuals in a group and grey lines connect neighbours. Neighbours have interactions, implemented as games of a specified kind, such as producer–scrounger, caller–satellite or hawk–dove. Groups consist of 21 individuals (*N* = 21), presented as points along the perimeter of a circle. Each individual in (*a*) is connected to two neighbours in the clockwise and two in the counterclockwise direction, so each has four neighbours (*K* = 4). In graph theory such a network is called a regular ring lattice. (*b*) Shows a so-called small-world network, obtained from the one in (*a*) through a ‘rewiring procedure’, as described by Watts & Strogatz [26]. The probability of rewiring a connection is *p*_rew_ = 0.1. (*c*) Illustration of the probabilities to produce and the actions taken (squares denote produce and triangles scrounge) for two individuals, shown colour coded, in the network in (*a*). (Online version in colour.)

Our general approach is to study reinforcement learning in games with two actions (behavioural options) for individuals in a group of size *N* that interact with neighbours in a social network. Figure 1 shows the kind of networks we study, with an illustration of two learning trajectories for a producer–scrounger game (see the electronic supplementary material for detailed descriptions of our methods).

The networks we use are either regular ring lattices (figure 1*a*) or small-world networks (figure 1*b*) [26,27]. The nodes of a network represent a group of *N* individuals and the network edges represent connections between a group member and the neighbours with which it interacts. For a ring lattice, each group member has *K* neighbours (figure 1*a*). A small-world network is obtained from a ring lattice by ‘rewiring’ some connections to a random, previously unconnected group member, with *p*_rew_ the probability of rewiring (figure 1*b*).

We use two implementations of reinforcement learning [25]: action–value learning and actor–critic learning. Action–value learning is a simple implementation of the classical Rescorla–Wagner model of conditioning [28], modified for instrumental conditioning. With two actions, for instance produce (P) and scrounge (S), a learning individual maintains and updates two estimates (e.g. *Q*_P_ and *Q*_S_) of the value (reward) of performing each action. As in the Rescorla–Wagner learning updates, the change in a value is the product of a learning rate (*α*) and the ‘surprise’, i.e. the difference between the actual perceived reward (*R*) and the currently estimated value (e.g. *Q*_P,*t*+1_ = *Q*_P *t*_ + *α*(*R*_*t*_ − *Q*_P*t*_) after performing action P in round *t*). The probability of choosing an action is a sigmoid (logistic) function of the difference in estimated values between that action and the alternative action, multiplied by a parameter *β* giving the sensitivity to differences in estimated values (e.g. a sigmoid function of *β*(*Q*_P_ − *Q*_S_); figure 1*c* illustrates action–value learning trajectories).

Actor–critic learning is a commonly used but more complex mechanism, which is related to so-called two-factor learning theory [29,30]. In this approach, the learning of values and the the updating of action preferences are coupled but separate psychological mechanisms. The expected value of a round, using the current action preferences, is updated using one learning rate (as in Rescorla–Wagner), and the action preferences, defined as the logit of the probability of choosing an action, are updated using another learning rate, but with the same value difference (the ‘surprise’). We show results from using the actor–critic learning rule in the electronic supplementary material, where the details of the rule are also described.

### (a) Games

For greater generality, we study three different two-action games with negative frequency dependence. In a round of the producer–scrounger game (with a total of *T* rounds), each group member chooses whether to produce or to scrounge. A producer has a probability *λ* of finding food. On finding food, the producer consumes an amount of value *V*_1_, after which scroungers can arrive, sharing the remaining amount *V*_2_ with the producer. We assume that scroungers come from the producer’s neighbours, but that a maximum of ${\hat{n}}_{S}$ scroungers can participate (if there are more available, ${\hat{n}}_{S}$ are randomly selected).

The caller–satellite game describes a group of males that can either call (C) to attract females, or to act as satellite (S) to neighbouring callers. They choose the action to use in each of a number *T* of rounds. Each caller has an effective call strength *s*. Because of interference (e.g. aggression) between callers, the call strength decreases with the number of neighbouring callers (*s* = 1 − *γ*_0_*k*_C_/*k*, where *k* is the number of neighbours and *k*_C_ is the number of these that call). The total number of females that are attracted to a group is proportional to the sum of the call strengths (with *f* the constant of proportionality). An attracted female approaches one of the callers with probability proportional to his call strength. If there are no satellites, the female mates with the caller, if there is a single satellite they each have a chance of 0.5 of mating, and if there are *k*_S_ satellite neighbours of the caller, each satellite has a probability 0.5/*k*_S_ of mating. This gives the caller an advantage in mating with the female. The reason can be that the female is trying to locate the caller and, possibly, that satellites interfere with each other when trying to intercept the female. The reward for mating is *V*_1_.

For the hawk–dove game, we assume that each group member has an expected number *T* of rounds (contests). Contestants are selected by first choosing a random group member and then a random opponent among the neighbours. Each contest is a standard hawk–dove game, with a benefit (reward) *V* of winning and a cost (penalty) *C* of losing a hawk–hawk fight. Details of this and the other games are found in the electronic supplementary material.

### (b) Learning simulations

Our results are based on individual-based simulations of learning in groups, typically 500 groups per case. As parameters we used *V*_1_ = 1, *V*_2_ = 3 and ${\hat{n}}_{S}=2$ for the producer–scrounger game; *γ*_0_ = 0.75, *f* = 2 and *V*_1_ = 2 for the caller–satellite game; and *V* = 1 and *C* = 2 for the hawk–dove game.

For action–value learning, we used *α* = 0.1 and *α* = 0.01 as learning rates for fast and slow learning, and *β* = 8 as the sensitivity to the difference in estimated values in the probability of choosing an action.

### (c) Description of polarization

We describe the degree of polarization of the individual learned action probabilities *p* in a group using a polarization index, $F=(\mathrm{Var}(p)/(\bar{\;p}(1-\bar{\;p}))$. The index is a normalized variance of the individual probabilities *p*. It is inspired by Wright’s fixation index as used in population genetics [31]. If all group members have the same *p*, *F* = 0, and if the probabilities are either 0 or 1, but vary between individuals, *F* = 1. With several groups, we average the index over groups.

To describe individual consistency over time, we use an autocorrelation, implemented as the correlation between the individual values of logit(*p*) at two points in time, as a function of the time difference (i.e. the time lag). This corresponds to the general approach of using a correlation of behaviour at two points in time to measure behavioural consistency [3,4].

## 3. Results

**Figure 2.**
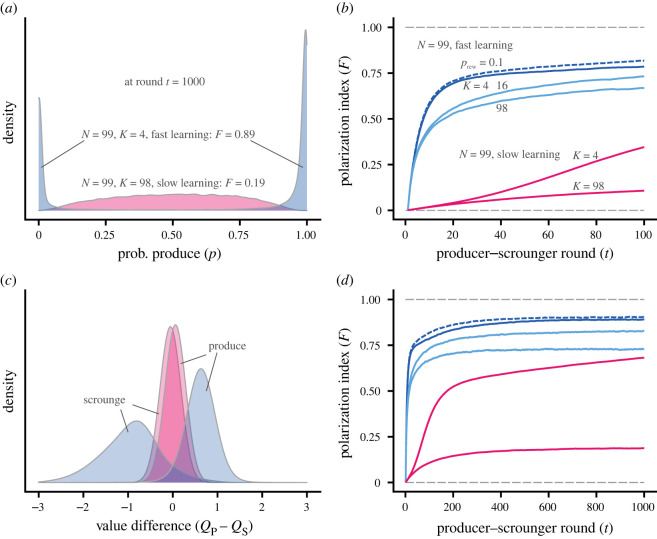
Behavioural polarization when there is action–value learning in a producer–scrounger game. Data are from 500 simulated groups per case and each group has N=99 members. (*a*) Distributions of the probability *p* to act as a producer after *t* = 1000 rounds of learning. Blue indicates a case where learning is fast (*α* = 0.10) and each individual is connected to *K* = 4 neighbours. Red is a case where learning is slow (*α* = 0.01) and all group members are connected (*K* = 98). The values of the group-mean polarization index *F* at *t* = 1000 for the two cases are indicated. (*b*) Change over time of the group-mean polarization index *F* for a number of cases. Blue curves show cases with fast learning (*α* = 0.10) and red cases with slow learning (*α* = 0.01), each labelled with the value of *K*. The dashed dark-blue line shows polarization in a small-world network obtained through rewiring (*p*_rew_ = 0.1) from the network illustrated by the dark-blue solid line, with *K* = 4. (*c*) Distributions of the difference between the estimated values of producing (*Q*_P_) and scrounging (*Q*_S_) after t=1000 rounds of learning, for the two cases in (*a*). The distributions are split according to an individual’s most recent action, scrounge or produce. (*d*) Same as (*b*) but over a greater number of rounds of learning. (Online version in colour.)

The types of networks and learning processes we model are illustrated in figure 1. With these kinds of social networks, but for a larger group size (*N* = 99), we simulated action–value learning for the producer–scrounger game (figure 2). For fast learning we find that substantial polarization into producers (P) and scroungers (S) emerges fairly rapidly, in particular for a small number of neighbours (figure 2*a*,*b*,*d*). For slow learning it takes longer for polarization to develop, but with a small number of neighbours, effects of frequency-dependence are strong, and polarization eventually reaches approximately the same level as for fast learning (figure 2*d* shows the first 1000 rounds). By contrast, with many neighbours and with all members connected, slow learning leads to a steady-state polarization with rather low value of the index *F* that we use to measure polarization (0 ≤ *F* ≤ 1; figure 2*a*,*d*). The explanation is that slow learning and many neighbours give rise to distributions of the difference in estimated values that overlap between group members that used P and S in the final round (reddish distributions in figure 2*c*), because learning averages long histories of nearly identical reward distributions. With fast learning, the estimated values represent learning over a smaller number of previous rounds, giving rise to distinct estimated value distributions between group members that used P and S in a given round.

The distributions of the difference *Q*_P_ − *Q*_S_ in figure 2*c* are split up according to the current action (P or S) used by an individual, and illustrate polarization. Thus, for *K* = 4 and fast learning (blue), the distributions for current producers and scroungers are separated, corresponding to strong polarization, whereas for *K* = 98 and slow learning (red) they are largely overlapping, corresponding to weak polarization.

**Figure 3.**
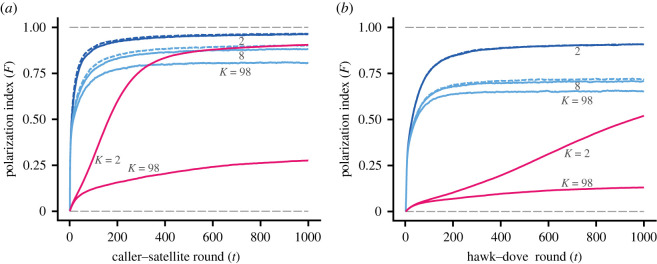
Behavioural polarization with action–value learning in caller–satellite and hawk–dove games. Data are from 500 simulated groups per case and each group has *N* = 99 members. (*a*) Change over time of the group-mean polarization index *F* for a number of cases of a caller–satellite game. Blue curves show cases with fast learning (*α* = 0.10) and red cases with slow learning (*α* = 0.01), each labelled with the number of neighbours *K*. The dashed lines shows polarization in small-world networks obtained through rewiring (*p*_rew_ = 0.1) from the networks with *K* = 2 and *K* = 8, respectively. (*b*) Same as (*a*) but for a hawk–dove game. (Online version in colour.)

Results for the caller–satellite game (figure 3*a*) and the hawk–dove game (figure 3*b*) were qualitatively similar to the producer–scrounger game, with rapid polarization for fast learning and a small number of neighbours. Small-world networks produced similar, and sometimes somewhat higher, polarization compared to the ring lattice they were constructed from (*p*_rew_ = 0.1;figures 2*b*,*d* and 3*a*,*b*).

**Figure 4.**
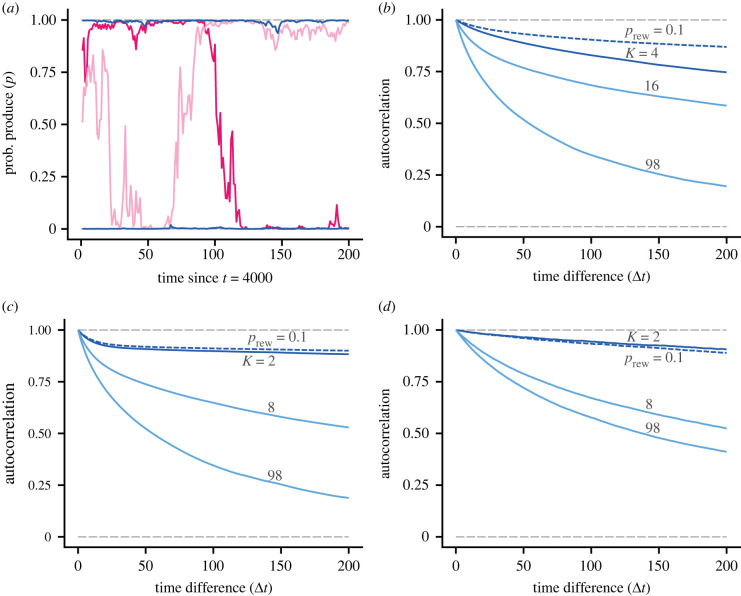
Illustration of behavioural consistency for different cases of social networks and games. Consistency tends to be higher in social networks with fewer neighbours. The group size is *N* = 99 for all cases. (*a*) Four examples of the individual probability *p* to act as a producer. The dark-blue curves show two examples with *K* = 4 neighbours, and the reddish curves show examples with *K* = 98 neighbours. In order to illustrate steady-state situations, the curves start at round *t* = 4000. (*b*) Autocorrelation for the logit of the probability to act as producer, for the fast-learning cases in figure 2*b*,*d* and using the same colour coding. In order to illustrate steady-state situations, the autocorrelations were computed from rounds between *t* = 4000 and *t* = 5000. (*c*) Autocorrelation for the logit of the probability to act as caller, for the fast-learning cases in figure 3*a*. (*d*) Autocorrelation for the logit of the probability to act as hawk, for the fast-learning cases in figure 3*b*. The autocorrelations are estimated from simulated individuals in five groups. (Online version in colour.)

Even though fast learning can give rise to pronounced polarization with many neighbours, the number of neighbours still has an important effect on individual behavioural consistency, as illustrated in figure 4*a*. We found higher temporal autocorrelation with smaller number of neighbours, for time lags of up to a few hundred rounds, for all three games (figure 4*b*,*c*,*d*). Our understanding is that this is caused by consistent differences between individuals in the expected rewards of actions, because of stronger effects of frequency dependence, in a similar way as was found by McNamara *et al.* [7] for smaller groups.

We repeated the learning simulations shown in figures 2–4 with actor–critic learning instead of action–value learning, and the results are shown in electronic supplementary material, figures S1–S3. Actor–critic learning shows some similarity to action–value learning in producing a somewhat faster build-up of polarization with a smaller number of neighbours in a social network. There is also a qualitative difference in that, after many rounds, actor–critic learning gives rise to extreme polarization, with very high consistency over time (electronic supplementary material, figure S3). Thus, after many rounds of actor–critic learning individuals develop strong action preferences, which limit their exploration of actions. This could be an unrealistic aspect of actor–critic learning, because reversal learning studies indicate that the algorithm takes longer to learn a reversal than is found in experiments [32]. A conclusion from a recent review [33] of the applicability of reinforcement learning algorithms, including action–value learning and actor–critic learning, is that both these have some support from neuroscience, but that more work is needed to develop a better understanding of reinforcement learning as implemented in real neural systems.

As a check of the robustness of our results, we simulated learning for the producer–scrounger game over a greater number of rounds and for a greater group size (electronic supplementary material, figure S4). Finally, similar distributions as in figure 2*c* but for the caller–satellite and hawk–dove games are shown in electronic supplementary material, figure S5.

## 4. Discussion

The general idea of behavioural specialization from frequency dependence [4,34], and in particular from frequency-dependent learning [8], forms the basis of our modelling approach. Experimental observations are consistent with such specialization through learning [11]. It is also experimentally established that learning can result in behavioural diversity rather than in uniformity and conformity [10]. These studies further show that learning to specialize happens after a fairly limited number of foraging events per individual, roughly corresponding to our model assumptions of fast learning.

The traditional approach in game theory in biology is to examine genetically determined strategies [6]. In small groups, the fact that an individual never encounters itself (in pairwise interactions) can influence whether a mixed ESS or a polymorphism of pure strategies is the expected outcome [35–37]. There are similar effects for learning in small groups. With negative frequency dependence, an individual’s preference for an action can cause others to learn to prefer a different action, and vice versa, and this is an explanation for behavioural specialization [7].

Theoretical analyses of learning in games, both in economics [38] and biology [24], tend to focus on the endpoints of learning, reached after many rounds of interaction. This allows investigation of correspondences between learning outcomes and game equilibria, such as ESSs, but it is important to consider possible limitations of the approach. In reality individuals might need to learn rather quickly, so that the consequences of learning after a fairly small number of rounds is the thing that matters. This should favour high rates of learning. As our results here illustrate, the rate of learning can have a qualitative influence on behavioural specialization (see also sections 5.2–5.5 in [19] for a discussion of effects of learning rates and the number of rounds). Recent experimental work in neuroscience further illustrates that learning is a complex process where individuals can adjust their learning rate, depending on how changeable the environment is likely to be [39].

Concerning social networks, there are observations on foraging in wild great tits (*Parus major*) indicating that individuals associate with a limited number of other birds [40]. For bats there are more detailed field observations of the number of producer–scrounger network neighbours [13], with individuals typically having only a handful of other group members that they predominantly interact with. There is also evidence that individuals show consistency over time in producer–scrounger relationships [12,41,42].

Our models assume that individuals do not differ in their inherent tendencies to prefer or learn about behavioural options. The reason for the assumption is to focus specifically on frequency-dependent learning, but it is likely to be an oversimplification of real situations. For instance, producer–scrounger studies have found that producing can correlate with better performance in a learning task [43], or that there are sex-differences in the tendency to produce [13,41,42]. It is even possible that consistency in the order in which individuals engage in an activity can influence which action they specialize on Dubois *et al.* [44]. Still, experiments show that individuals can change specialization in a new social environment [12].

Less is known about frequency-dependent learning of caller–satellite specialization in the field. Observations indicate that males use calls to assess the size or strength of neighbouring males in anurans and that this influences their behaviour [20,21]. There is thus the possibility that learning about the social environment plays a role in behavioural specialization, and it is also likely that variation in individual characteristics has a considerable influence on which behaviour is learnt.

As mentioned, our hawk–dove model could be a simple starting point for modelling of social dominance in small groups of individuals with limited individual recognition. This might be the case for males in some species of crickets [22,23,45] but, again, individual characteristics relating to fighting ability are likely to be important in these situations.

In conclusion, our results show that frequency-dependent learning can give rise to behavioural specialization in a social network. We have identified the number of network neighbours and the rate of learning as potentially important for the speed at which specialization emerges in a group, and possibly also for the strength of polarization and the consistency of behaviour over time. Further experimental work investigating these aspect would improve our understanding of the factors behind behavioural specialization.

## Data Availability

C++ source code for the individual-based simulations is available at GitHub, together with instructions for compilation on a Linux operating system: https://github.com/oleimar/behavspec. Electronic supplementary material is available online [46].
